# Health disparities in preterm births

**DOI:** 10.3389/fpubh.2023.1275776

**Published:** 2023-12-15

**Authors:** Judy Brown, Xiaolin Chang, Adam Matson, Shabnam Lainwala, Ming-Hui Chen, Xiaomei Cong, Sharon G. Casavant

**Affiliations:** ^1^Institute for Systems Genomics, University of Connecticut, Storrs, CT, United States; ^2^School of Nursing, University of Connecticut, Storrs, CT, United States; ^3^Department of Statistics, University of Connecticut, Storrs, CT, United States; ^4^Division of Neonatology, Connecticut Children’s Medical Center, Hartford, CT, United States; ^5^Department of Pediatrics, School of Medicine, University of Connecticut, Farmington, CT, United States; ^6^Yale University School of Nursing, Orange, CT, United States

**Keywords:** health inequities, preterm birth, neurodevelopment, pain, social determinants of health

## Abstract

**Introduction:**

Black African American (B/AA) women have a 2-fold to 3-fold elevated risk compared with non-Hispanic White (W) women for preterm birth. Further, preterm birth is the leading cause of mortality among B/AA infants, and among survivors, preterm infant adverse health outcomes occur disproportionately in B/AA infants. Racial inequities in maternal and infant health continue to pose a public health crisis despite the discovery >100 years ago. The purpose of this study was to expand on reported preterm infant outcome disparities. A life-course approach, accumulation of lifelong stress, including discrimination, may explain social factors causing preterm birth rate and outcome inequities in B/AA mothers.

**Methods:**

Anthropometric measures and clinical treatment information for 197 consented participants were milled from electronic health records across 4 years. The Neonatal Infant Stressor Scale was used to tally acute and chronic painful/stressful procedures. Neurobehavioral differences were investigated using the Neonatal Intensive Care Unit (NICU) Network Neurobehavioral Scale.

**Results:**

B/AA mothers gave birth to preterm infants earlier than W mothers. NICU hospitalization stays were extended more than 2 weeks for the significantly smaller B/AA preterm infants in comparison to the age-matched W preterm infants. A higher number of chronic lifesaving procedures with demonstrated altered stress response patterns were recorded for B/AA preterm infants.

**Discussion:**

This cross-sectional analysis of preterm birth rates and preterm infant developmental and neurodevelopmental outcomes are presented in the context of NICU stress and pain, with attendant implications for infant mortality and future health disparities. Preterm birth rate and outcome inequities further support the need to develop interventions and policies that will reduce the impact of discrimination and improve social determinants of health for Black, Indigenous, and other People of Color.

## Introduction

Preterm births are a significant healthcare concern worldwide, with an estimated 15 million infants born preterm (<37 weeks’ gestational age [GA]) globally each year.^1^ Preterm births also represent the second most common cause of infant mortality in the US population overall (1). Unfortunately, national statistical systems reports show an increased incidence of preterm births among live births from 9.8 to 10.5% between 2011 to 2021.^2^ There is further evidence among data for stark racial disparities in preterm birth risks and infant health outcomes.^3^ The preterm birth rate for Black/African Americans (B/AA) in the United States is 52% higher than that of all other races.^4^ Preterm birth is the leading cause of infant mortality among B/AA (2) and for the preterm infants surviving infant mortality, reaching their first birthday, there is a high risk for infections, developmental difficulties, breathing problems, and lifelong health complications. Social determinants of health, including neighborhood environmental exposures and built environment, experiences of discrimination and limited access to quality health care can explain many pregnancy disparities (3). However, although the first report of racial differences in pregnancy outcomes was made 125 years ago (4) the outreach and funding priorities of health organizations remain strategies to reduce preterm births as a public health goal.^5^ At the same time, researchers continue to seek answers to explain disparity determinants.

Being born preterm is a perturbation in development; any additional disturbance is deemed a stress event. Preterm infants are hospitalized in neonatal intensive care units (NICU) for many reasons and often extensive periods, undergoing a mean of 17.3 painful procedures in the first 2 weeks of life (5). Pain and stress are inflicted on infants simply as part of routine, lifesaving care (6). Infants in the NICU, especially the extremely preterm (<28 weeks’ GA), the very pre-term (28–32 weeks’ GA), are likely to receive invasive interventions with higher pain intensity (e.g., gastric tube insertion and blood collection), however repositioning, diaper changes and bathing, while less invasive, are still stressful (7). Preterm infants have a heightened sensitivity to tactile and nociceptive (sensory) neuronal input making it challenging to distinguish between noxious and non-noxious stimuli (8). The number and duration of repeated stress/pain-inducing events harm the preterm infants’ rapidly developing central nervous and neuroendocrine systems, (9, 10) with associated long-term pathological outcomes recognized as poor neurodevelopment, autonomic nervous system dysfunction, and psychiatric or behavioral disorders (11).

Communication with infants about perceived pain is not realizable given crying may also indicate other conditions (hunger, tiredness), and facial expression interpretation is highly subjective. Behavioral, physiological, and biochemical markers are thus adopted as indicators of pain. Biochemically, stressful/painful procedures are accompanied by increased cortisol levels. An infant’s cortisol levels are higher during the early days in the NICU (12) when an infant is still adapting to acute stressors, a period called allostasis (13). Tracking physiological exposures to pain and stress is collected on the Neonatal Infant Stressor Scale (14). Acute pain procedures resolve relatively quickly, including, for example, peripheral venous catheter placement, blood collection via heel stick, or endotracheal tube placement. Longer-term procedures such as mechanical ventilation, supplemental oxygen administration, or central catheter placement are tallied as chronic pain procedures. Early detection of neurobehavioral delays may be identified by the use of standardized assessments such as the NICU Network Neurobehavioral Scale (NNNS) (15).

B/AA preterm infants are born at an earlier gestational age than preterm infants of other races; as such, B/AA preterm infants are likely exposed to more painful/invasive procedures in the NICU (16, 17). We have also reported B/AA preterm infant outcome disparities in gestational age at birth (*p* < 0.005) and in birth weight, length, and head circumference (*p* < 0.0001). Growing evidence suggests that life-course epidemiology, including racism-related stress over time, may contribute to health inequities in preterm birth rate and infant mortality (18–22). To expand the data and knowledge base for preterm infant studies, we conducted a cross-sectional study of the developmental and neurodevelopmental outcomes of B/AA preterm infants resulting from stressful and painful procedures in the NICU. Stressful/painful procedures are quantified using the Neonatal Infant Stressor Scale, categorizing procedures as “acute” or “chronic” (14). Examples of highly stressful/painful acute procedures include surgery, insertion of umbilical arterial or venous central catheters, multiple intravenous catheter placement and eye exams for retinopathy of prematurity. Examples of highly stressful/painful chronic procedures include chest tube *in situ*, conventional ventilation *in situ*, nasopharyngeal suctioning, replogle irrigation and *in situ* and systemic infections such as sepsis, meningitis or pneumonia (14). We proposed that given earlier gestational ages, and lower birthweights of B/AA preterm infants, chronic painful procedures would outnumber acute pain/stress events. Significantly higher incidences of diabetes, hypertension, coronary artery disease, and stroke have been explained by race in B/AA adults (23). However, improvement in neonatal trauma-informed care necessitates steps toward understanding the *impact of disparities* to reduce subsequent prolonged adverse health outcomes for preterm infants.

## Methods

### Participants

This human participant research study was reviewed and approved by Connecticut Children’s Medical Center Institutional Review Board (IRB #16–001). The inclusion criteria were infants with GA at ≤32 weeks or birth weight less than 1,500 grams, and delivery during 2016–2020. Premature infants were cared for at either the urban (Hartford) or suburban (Farmington) locations of Connecticut Children’s Medical Center NICU departments. Exclusion criteria included major brain lesions (intraventricular hemorrhage >Grade 2, periventricular leukomalacia), neurosensorial deficits (retinopathy of prematurity >Stage 2), and a clinical history of genetic syndromes and/or major malformations. Informed consent was provided verbally in English or Spanish. Parents/legal guardians provided written informed consent on behalf of the infant to participate in the study. Deidentified electronic health record data, including demographic and clinical course of treatment, were entered into a password-secured REDCap database.

### Measurements and assessments

The Neonatal Infant Stressor Scale (NISS) is the standardized instrument (14, 16) for tabulating the number and types of distress events an infant is exposed to during a NICU stay. The number of marked acute stressor columns out of 44 possible acute stressors is added to the marked columns out of 24 chronic stressors (Supplementary Table 1) for a cumulative stress score daily.

Before the creation of the NICU Network Neurobehavioral Scale (NNNS) (24), Pretchel and Beintema (25) posited that for valid interpretation of the standardized NISS measure, observational and clinical data must be collected during specific physical and behavioral infant conditions or “states”. The NISS (25) and, later, a neonatal intensive care unit-specific measure, the NICU Network Neurobehavioral Scale (24), defined infants attending and orienting themselves and producing organized motor acts as neurodevelopmentally proficient (24). As neurological scales evolved, additional criteria were added, e.g., orientation, habituation, tone, and reflexes (26). Practitioners and nurses could evaluate the items on the neurological scales to gauge an infant’s neurological status, guided by the fact that an intact brain can organize states. In contrast, an impaired brain cannot organize these states (25). Consolidation of neurodevelopment criteria was the impetus for developing the NICU Network Neurobehavioral Scale (NNNS) (27) The NNNS is a standardized neurobehavioral assessment with good psychometric properties (internal and concurrent validity) used in the NICU for early detection of neurobehavioral challenges in at-risk populations (15, 28). The assessment records 13 states: habituation, attention, arousal, regulation, handling procedures, quality of movement, excitability, lethargy, nonoptimal reflexes, asymmetric reflexes, hypertonicity, hypotonicity, and stress/abstinence (28). Measurements for the NNNS are plotted against the theoretical scale to identify tendency and percentile values (29). The NNNS was utilized on inpatient preterm infants observed by a trained assessor at 36–37 weeks postmenstrual age. Postmenstrual age considers the baby’s gestational age at birth plus the postnatal age (days of life) (30).

### Statistical analyses

Descriptive statistics were calculated for quantitative variables, including mean, median, and interquartile range. Qualitative variables used to describe characteristics were compared using frequency calculations. A Wilcoxon test/*t*-test for numerical variables and a Pearson chi-square test/Fisher exact test for categorical variables were performed to assess differences between groups. Data was cleaned, processed, and analyzed using R (version 4.2.0) (31). Statistical calculations were considered significant at *p* = 0.05.

## Results

**Figure 1:**
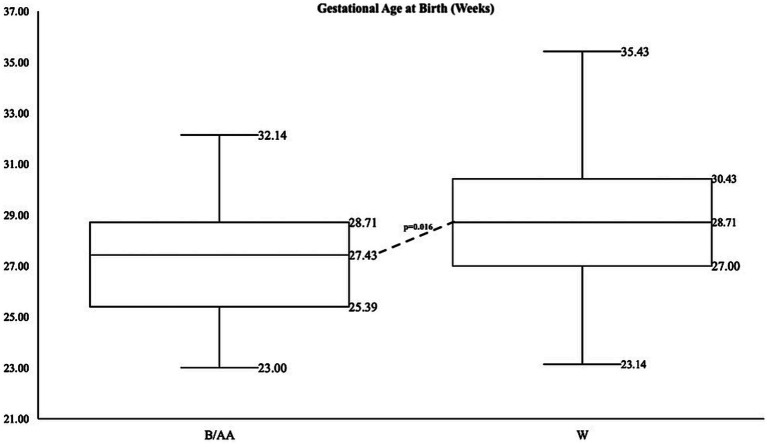
Summary statistics for gestational age by race with data points on box whisker plots from lowest value (minimum), first quartile, median, third quartile, and maximum value for B/AA infants on the left and W infants on the right.

A limited number of consented patients were of varying races, as identified by parents or legal guardians and abstracted from the electronic health record. The number of B/AA infants in each of the GA categories (<28 weeks, 28–32 weeks, >32 weeks) was statistically significantly different than the numbers of W infants in the same respective GA categories (*p* = 0.016). A Fisher’s exact test for count data indicted that 31 B/AA infants were born extremely preterm, 19 were born very preterm and 1 was born in the preterm GA category. The numbers for W infants were 55, 86, and 5, respectively. Black/African American non-Hispanic (hereto referred to as B/AA) or White non-Hispanic (now referred to as W) preterm infants. B/AA mothers gave birth to preterm infants earlier than W mothers, with a median GA at birth of 27.2 weeks and 28.6 weeks, respectively, (*p* < 0.001) (Figure 1). B/AA preterm infants were born smaller with lower birthweight, birth length and head circumference (Table 1). The demographic data revealed a disparity in the length of hospital stay by race (*p* = 0.0289) (Table 1).

**Table 1:** B/AA preterm infants are smaller and have extended hospital stays versus W preterm infants.

	Black (*n* = 51)	White (*n* = 146)	
Indicator	Median [1^st^ quartile; 3^rd^ quartile]		Value of *p*
Birth weight (g)	850.0 [701.3;1098.0]	1125.0 [860.0;1395.0]	0.000
Birth length (cm)	34.3 [33.0;37.0]	37.2 [34.5;40.0]	0.000
Birth head circumference (cm)	24.0 [22.5;25.5]	26.0 [24.0;27.5]	0.000
Hospital stay (days)	91.5 [67.0;118.0]	75.0 [53.0;102.0]	0.029

**Figure 2:**
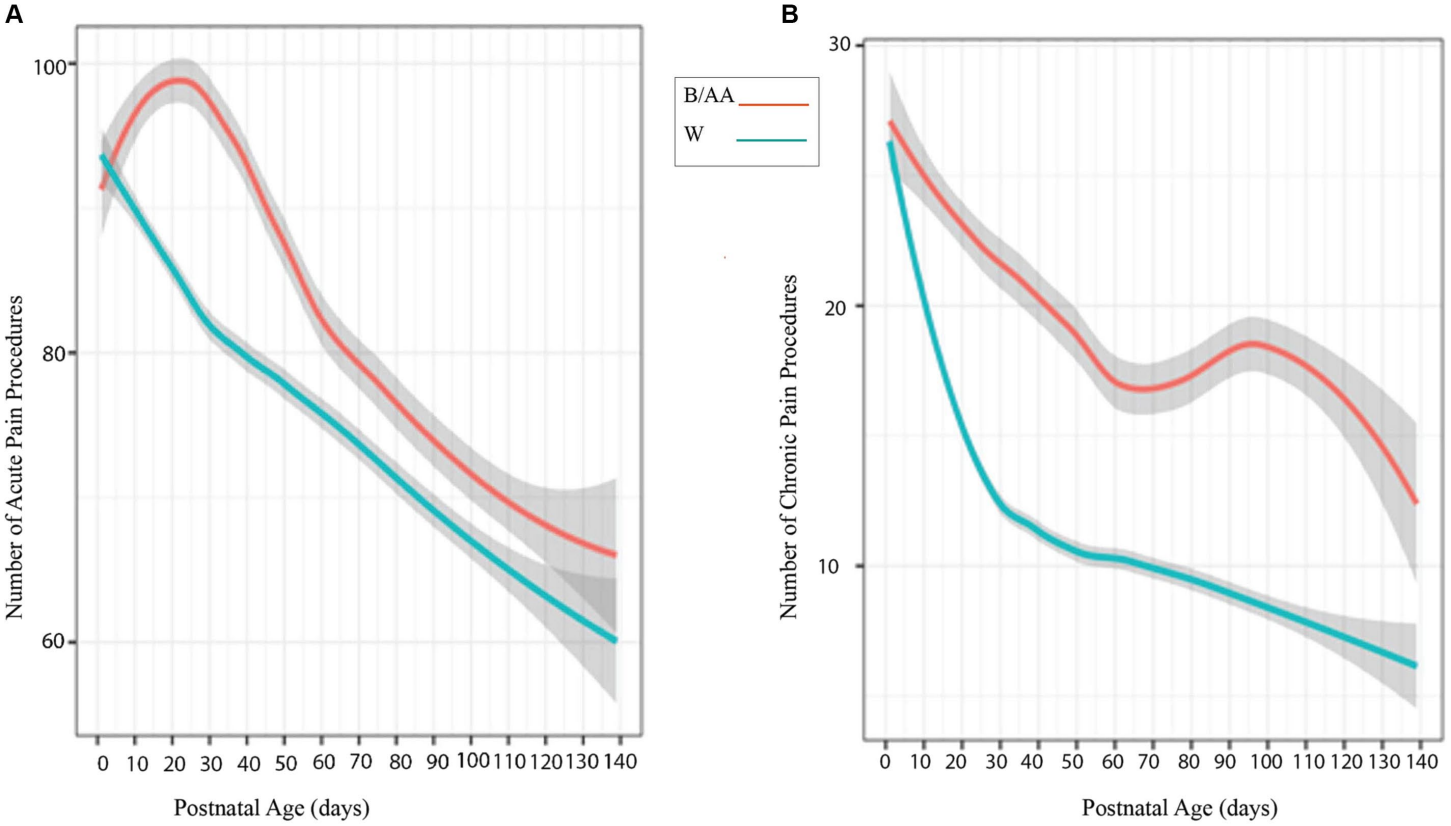
**(A)** Looking at mean over time, the number of Neonatal Infant Stressors for B/AA preterm infants (pink/top line) and W preterm infants (blue/bottom line) over postnatal age by day during the NICU stay. B/AA preterm infants experienced more acute pain procedures (n.s.) and chronic painful/stressful procedures (*p* = 0.01) than the W preterm infants **(B)**.

Postnatal ages and numbers and types of acute and chronic painful/stressful procedures (Supplementary Table 1) were retrieved from patient medical records for significance calculations between B/AA and W infants. The number of acute painful/stressful procedures recorded on the Neonatal Infant Stressor Scale (NISS) over post-natal age (PNA) by day during preterm infant stays in the NICU produces a trendline evident in Figure 2A, that although not statistically significant, shows B/AA preterm infants experienced more acute pain experiences over time. A higher number of chronic painful/stressful procedures over PNA by day, with statistical significance, was identified for B/AA infants (Figure 2B). There was no statistically significant variation for 12 of the 13 NNNS scales evaluated across the B/AA and W infant populations. A statistically significant difference was identified for the NNNS scale measurement for handling between the B/AA and W infant populations (*p* = 0.036) (Supplementary Figure 1).

## Discussion

Preterm birth rates and infant mortality statistics document evidence of continued racial disparities, making preterm birth a significant global health concern. Evidence of inequities resulting from preterm birth include lower gestational age at birth and lower anthropometric measures in B/AA non-Hispanic infants compared to W non-Hispanic. Infants with a lower gestational age at birth will lack maturity in crucial organ systems, including the lungs, brain, and gastrointestinal tract. Very early GA births will thus have a higher likelihood of accompanying medical complications needing interventions for the survivability of the preterm infant. Mean event trend lines of the Neonatal Infant Stressor Scale (NISS) data provide visual confirmation of the proposal that longer NICU stays coincide with a higher number of pain procedures (Figure 2). Separation of the acute stressor trend line (n.s.) and the significant separation (*p* = 0.01) between the chronic pain trend lines for B/AA preterm infants and W preterm infants (Figure 2) supports the racial disparity in gestational age at birth (Table 1) and correlates with the statistically significant difference in length of hospital stay between the B/AA and W preterm infants.

Painful/stressful procedures trigger the hypothalamic–pituitary–adrenal (HPA) axis (12) to release cortisol with each procedure. However, the concept of allostasis and allostatic load must also be introduced (32). McEwen identified four stages that give rise to allostatic load, (the tipping point where the body is not responding appropriately) and their potential pathological impact. Those stages are (1) Normal allostatic response to a stressor (in this case, release of glucocorticoids) where the response is maintained for an appropriate amount of time and then inactivates; (2) Undergoing repeated “hits” from multiple stressors; (3) Lack of adaption leading to a delay in inactivation; and, (4) Inability to mount an adequate response which leads to compensatory hyperactivity of other mediators (i.e., inadequate secretion of glucocorticoids leading to increased concentration of cytokines typically regulated by glucocorticoids) (6). Therefore, once the body reaches stage four, we expect to see an absence of glucocorticoids. The “nhandle” value of the NNNS measurement may be used to reflect allostatic load, the scenario of an inadequate HPA axis response. Neurotypical infants require ~6 maneuvers to be soothed either by swaddling, rocking, walking, pacifier, or other methods (27). In our study, a median handling value of 0.25 (Supplementary Figure 1) shows that the B/AA infants require very few maneuvers to be soothed.

Although limited by lower participation numbers of B/AA women, our findings support disparities among B/AA preterm infant births and outcomes (3, 17, 33–38). We continue to expand recruiting efforts for a more diverse representation of mothers and infants as our research moves toward clarifying variables that may lead to B/AA pregnancy disparities. Longer NICU stays exacerbate the number and types of painful/stressful procedures for the infant but may also stress the mother-preterm infant relationship. A more extended stay has the potential to strain the mother-preterm infant relationship due to separation and the delicate yet dynamic interplay of oxytocin (the bonding hormone) between mother and infant (39). Maternal mental health may also be harmed when the bonding relationship is disrupted (40). Extended hospital stays with concomitant higher preterm infant care bills impart additional maternal stress. Of the live births in the United States in 2021, 10.5% (383,979) were preterm, with an annual price tag of $25 billion or $656,286 per preterm infant.^6^ Hospital expenditures for preterm infant care differ depending on gestational age and weight at birth. The average costs from 2008 to 2016 for late preterm births were $76,153; for low birthweight preterm infants, $114,437; and extremely preterm infants at 24 weeks GA were $603,778 (41).

Provision of colostrum and breastmilk is associated with long-term positive health outcomes, including risk reduction for infectious diseases, cancer, autoimmune disorders, high blood pressure, and more. B/AA communities face heightened risks for diabetes, infant obesity, high blood pressure, and ovarian and breast cancer. Maternal racial disparity, in part, also influences breastmilk provision and milk nutrient composition (42, 43). Support for mothers providing breast milk significantly affects the decision to start and maintain breastfeeding in B/AA women (44, 45). Insufficient milk delivery accounts for some sluggish weight gain and differences in anthropometric measures in B/AA preterm infants compared to W preterm infants during hospitalization and hospital discharge (17, 46). Breastmilk support is an area of investigation that may be important in reducing B/AA preterm infant health disparities. Most very preterm or extremely preterm infants cannot produce the sequential processes of swallowing and breathing simultaneously to eat by mouth until closer to term GA. Therefore, nutrition is delivered either via central line catheterization or placement of a naso- or oro-gastric tube, both painful and stressful.

**Figure 3:**
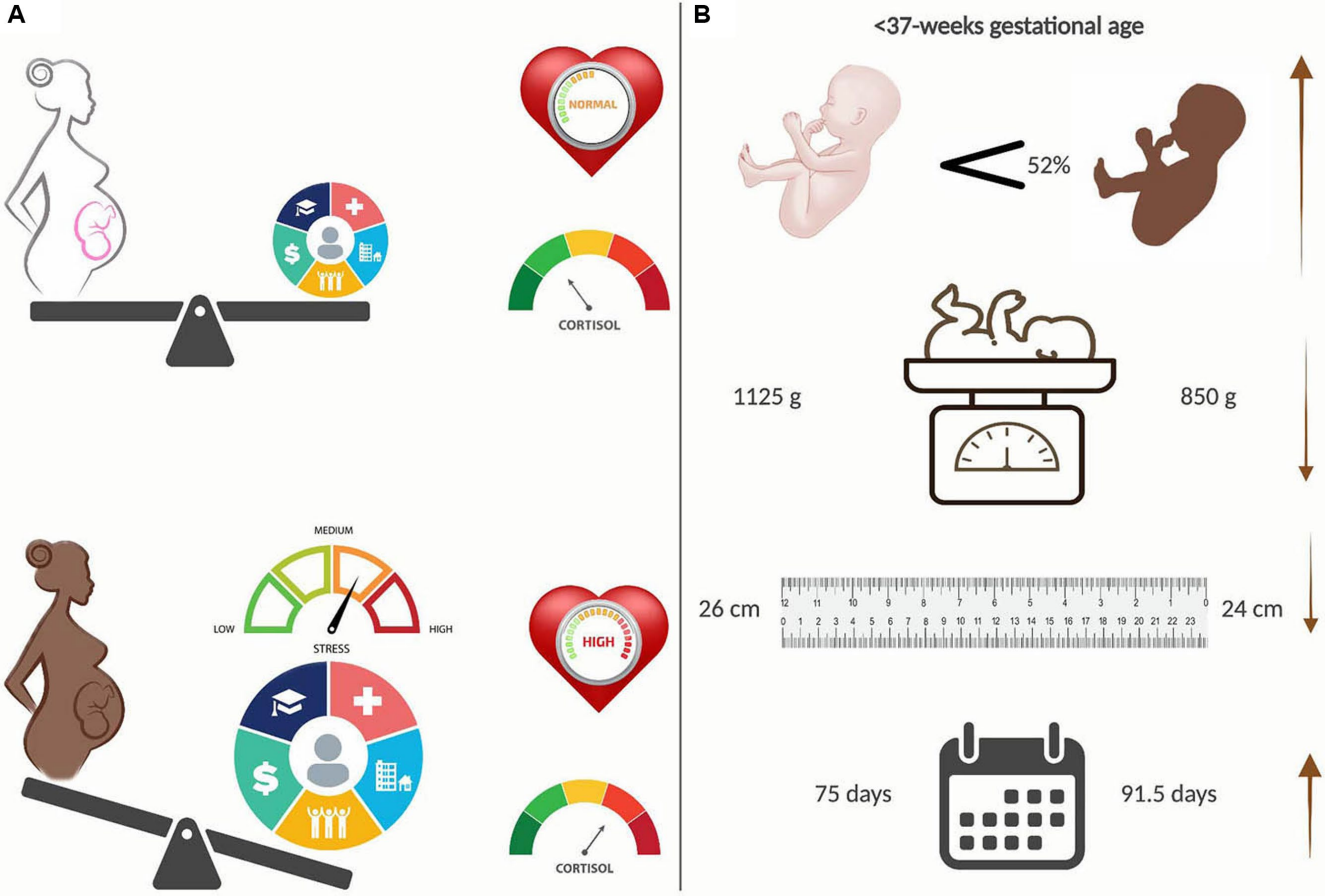
Racial disparities in preterm births. **(A)** Black/African American (B/AA) women experience disproportionately heavier stress than their White (W) counterparts, primarily because of Social Determinants of Health (SDOH), leading to increased measures of cardiometabolic syndromes, e.g. blood pressure and cortisol levels. **(B)** B/AA preterm babies are born 52 percent more often, are significantly smaller and have longer hospital stays than W preterm babies. Created with BioRender.com.

The data presented within adds to the growing field of study about racial health inequities, specifically disparities in preterm birth rates and outcomes (34). Research has moved toward efforts to identify the causative factors of recognized differences. Discerning the equation to explain disparities in preterm birth rates and outcomes for B/AA women requires, at minimum, the inclusion of NICU standards, trauma-informed chronic care practices, and social determinants of health. We propose life-course stress as the overarching theme for disparities in social health determinants. B/AA women disproportionately experience stress with measurable clinical outcomes of increased cardiometabolic syndromes (Figure 3A) and an increased incidence of preterm births (Figure 3B). Preterm infants of B/AA women are born earlier and have accompanying findings of lower birth weight, smaller length, and more extended hospital stays (Figure 3B).

Furthermore, preterm infants with associated delayed development will have increased exposure to more frequent and chronic pain procedures. Improving health equity is crucial to reducing the number of preterm births and their challenging courses of treatment, often resulting in adverse developmental outcomes. While this study highlights significant health inequities in preterm birth rates and outcomes, further research is needed to explore potential interventions to address social determinants of health that contribute to these inequities and to offer recommendations on how healthcare providers and policymakers might improve outcomes for preterm infants.

## Data availability statement

The original contributions presented in the study are included in the article/Supplementary material, further inquiries can be directed to the corresponding author.

## Ethics statement

The studies involving humans were approved by Connecticut Children’s Medical Center Institutional Review Board. The studies were conducted in accordance with the local legislation and institutional requirements. Written informed consent for participation in this study was provided by the participants’ legal guardians/next of kin.

## Author contributions

JB: Conceptualization, Writing – original draft, Writing – review & editing, Visualization. XCh: Writing – review & editing, Formal analysis. AM: Data curation, Writing – review & editing. SL: Writing – review & editing, Data curation. M-HC: Supervision, Writing – review & editing, Methodology, Resources, Software. XCo: Data curation, Supervision, Writing – review & editing. SC: Conceptualization, Investigation, Writing – original draft, Writing – review & editing.

## Footnotes

^1^WHO. 2022. “Fact Sheet: Preterm Birth.” accessed January 10. https://www.who.int/news-room/fact-sheets/detail/preterm-birth.

^2^Dimes Mo. 2022 Report Card [July 1, 2023]. Available from: https://www.marchofdimes.org/sites/default/files/2022-11/2022-MarchofDimes-ReportCard-UnitedStates.pdf.

^3^National Vital Statistics System - Natality (NVSS-N) 2021. Available from: https://health.gov/healthypeople/objectives-and-data/data-sources-and-methods/data-sources/national-vital-statistics-system-natality-nvss-n.

^4^Dimes Mo. 2022 Report Card [July 1, 2023]. Available from: https://www.marchofdimes.org/sites/default/files/2022-11/2022-MarchofDimes-ReportCard-UnitedStates.pdf.

^5^National Vital Statistics System - Natality (NVSS-N) 2021. Available from: https://health.gov/healthypeople/objectives-and-data/data-sources-and-methods/data-sources/national-vital-statistics-system-natality-nvss.

^6^https://www.marchofdimes.org/peristats/reports/united-states/prematurity-profile

## Supplementary material

Supplementary Figure 1 NNNS handling scores. Summary statistics for NNNS handling score with data points on box whisker plots from lowest value (minimum), first quartile, median, mean (x), third quartile, and maximum value for B/AA infants on the left and W infants on the right. Dashed line between median scores represents significant difference at *p* = 0.036.
