# Supplementary figures and images for: Health disparities in preterm births
Source: Front Public Health. 2023 Dec 15;11:1275776. doi: 10.3389/fpubh.2023.1275776 (PMC10757361; doi:10.3389/fpubh.2023.1275776)

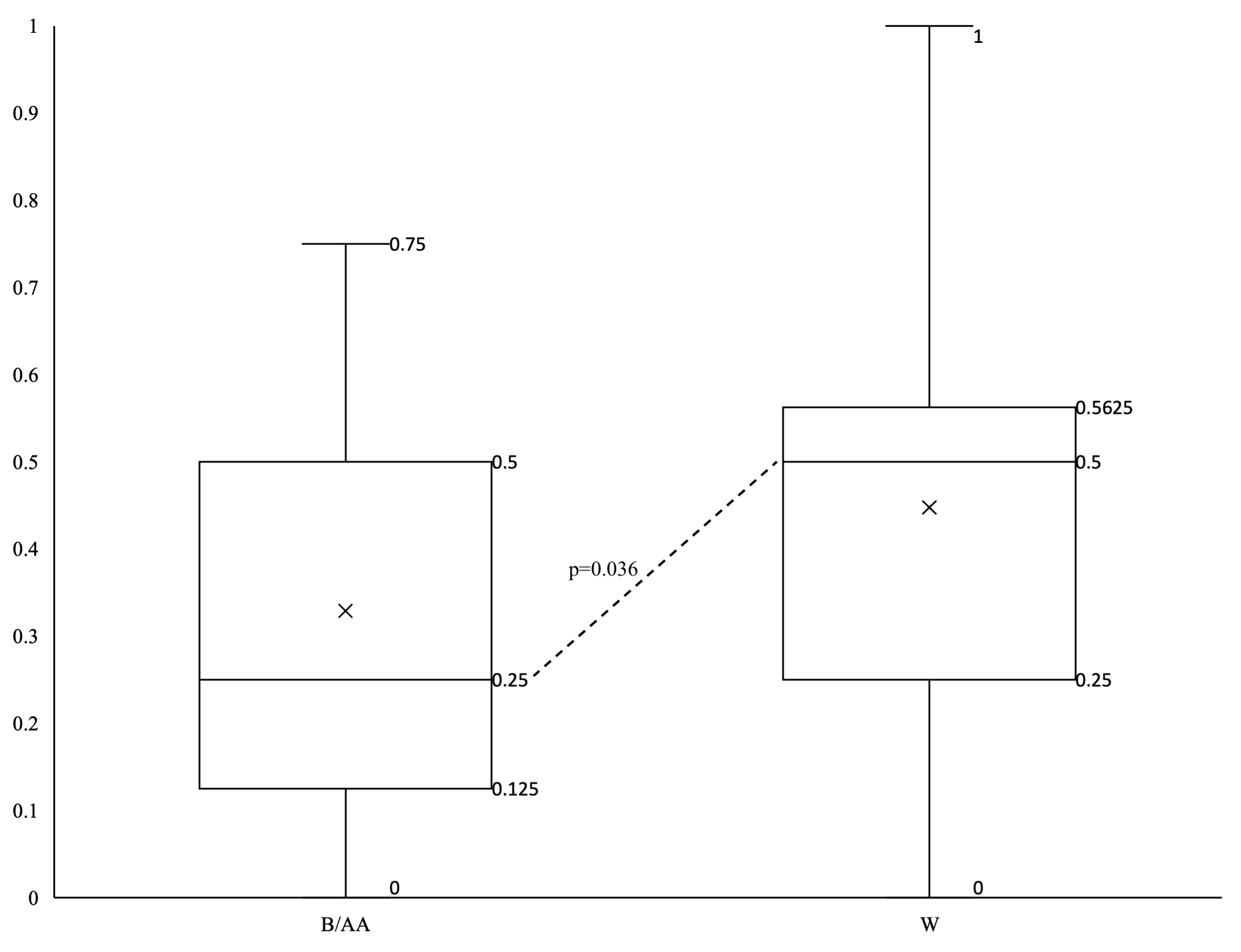

Supplement: Supplementary Figure 1 — NNNS handling scores. Summary statistics for NNNS handling score with data points on box whisker plots from lowest value (minimum), first quartile, median, mean (x), third quartile, and maximum value for B/AA infants on the left and W infants on the right. Dashed line between median scores represents significant difference at p = 0.036. [file Image_1.jpg]
