# Supplementary material for: Health disparities in preterm births
Source: Front Public Health. 2023 Dec 15;11:1275776. doi: 10.3389/fpubh.2023.1275776 (PMC10757361; doi:10.3389/fpubh.2023.1275776)
Supplement: Supplementary file 2 [file Table_1.docx]

Supplemental Table 1. Acute and Chronic Stressors on Neonatal Infant Stressor Scale Decreasing in Pain/Stress Intensity

| **Acute Stressor** | **Chronic Stressor** |
| --- | --- |
| 1. Multiple intravenous (IV) catheter insertions | 1. Gastroschesis recovery |
| 1. Umbilical arterial central catheter insertion (UAC) | 1. Recovery from surgery |
| 1. Umbilical venous central catheter insertion (UVC) | 1. Chest tube in situ |
| 1. Surgery | 1. Systemic infection |
| 1. Retinopathy of prematurity eye exam | 1. High flow oxygen supplementation extended |
| 1. Intubation | 1. High flow oxygen with sedation |
| 1. Chest tube insertion | 1. Mechanical ventilation extended |
| 1. Blood collection via arterial stick | 1. Mechanical ventilation with sedation |
| 1. Blood collection via heel stick | 1. CPAP extended |
| 1. Single IV insertion | 1. High humidity nasal cannula extended |
| 1. IV tourniquet | 1. Nil per os extended |
| 1. Peripherally inserted central catheter (PICC) | 1. Local infection |
| 1. PICC tourniquet | 1. IV in situ |
| 1. UAC removal | 1. PICC in situ |
| 1. UVC removal | 1. UAC in situ |
| 1. Circumcision | 1. UVC in situ |
| 1. Lumbar puncture | 1. Urinary catheter in situ |
| 1. Urinary catheter | 1. Replogle in situ |
| 1. Extubated | 1. Low flow nasal cannula extended |
| 1. Chest tube removal | 1. Intranasal oxygen extended |
| 1. Nasogastric/orogastric tube insertion | 1. Phototherapy |
| 1. Suction endotracheal tube | 1. NG tube in situ |
| 1. Replogle insertion | 1. OG tube in situ |
| 1. Systemic infection | 1. Environmental stimulation |
| 1. IV removal |  |
| 1. PICC removal |  |
| 1. PICC dressing change |  |
| 1. CPAP manipulation |  |
| 1. Remove replogle |  |
| 1. Diaper change/Position change |  |
| 1. Incubator change |  |
| 1. Change monitor leads/Remove tape |  |
| 1. Weigh |  |
| 1. Nil per os |  |
| 1. Abdominal x-ray/Chest x-ray |  |
| 1. Local infection |  |
| 1. IV flush |  |
| 1. Nasal suction |  |
| 1. Replogle irrigation |  |
| 1. Chest physiotherapy |  |
| 1. Bathing |  |
| 1. Learning to breast/bottle feed |  |
| 1. Echocardiogram/Ultrasound |  |
| 1. CT Scan/MRI |  |
